# Breaking the patientification process - through co-creation of care, using old arctic survival knowledge

**DOI:** 10.1080/17482631.2021.1926052

**Published:** 2021-05-11

**Authors:** Ulrika Sandén, Lars Harrysson, Hans Thulesius, Fredrik Nilsson

**Affiliations:** a Department of Design Sciences, Lund University, Lund, Sweden; b School of Social Work, Lund University, Lund, Sweden; c Department of Medicine and Optometry, Linnaeus University, Växjö, Sweden

**Keywords:** Cancer, design thinking, health, hope, identity, innovation, patient perspective, rehabilitation, waiting, co-creation

## Abstract

Purpose: Cancer research and connected innovation processes often lack a major component; patient participation. We revisit three studies (a-c) in order to explore how Momentary contentment theory may be used to improve patient participation and psychosocial health.

Method: We revisited data from the initial (a) classic grounded theory study on Momentary contentment, based on four years of observation and 14 interviews. It explains a way of dealing with life close to death and morbidity. In the imminence of danger the studied culture resembles the context of cancer patients. The two following studies used focus group interviews with (b) 19 cancer patients and (c) 17 relatives of cancer patients in southern Sweden.

Results: We suggest a process where cancer patients are taught to be submissive and that the support they receive from health providers may be counterproductive to contentment; a patientification process. We present alternative ways for people to handle issues such as hope, waiting, knowledge gaps and healthcare navigation while living with cancer. We introduce an alternative to patientification and passive patients where active patients create their own safety and truly participates in their care.Conclusions: We propose clinical studies to introduce such a shift from patentification to co-creation of care.

## Introduction

Individualized, precision medicine is widely acknowledged in cancer treatment and research. At the same time cancer care overlooks incorporation of patient values and objective evidence into decision-making (Hirsch & Abernethy, 2013). Historically, the patient has been seen as a passive recipient of someone else’s actions (Gunnarson, 2016; Sandén, 2017). Even if patients have been more involved in their care during recent decades (Bate & Robert, 2006) our informants still express a need for further developing the involvement of patients and loved ones (Sandén et al., 2017, 2019). Patient passivity contradicts the view of an innovative person using their tacit knowledge and experience to create order in a world of illness.

Momentary contentment theory (Sandén, 2017; Sandén, Harrysson et al., 2015) explains how old survival knowledge in a remote arctic village has been transformed from historically being a matter of life and death to contributing to increased contentment in current society. The theory is based on safety-enhancing activities where inclusion, helpfulness and acceptance are central parts of the culture. We have previously shown a theoretical fit between momentary contentment and a cancer patient context (Sandén et al., 2017).

Momentary contentment theory shows how a history of isolation, harsh climate, and risky occupations have created a proximity to death and need for security. The sense of control, apparent in many health theories (Antonovsky, 1996; Bandura, 1997), is in momentary contentment theory exchanged by an acceptance of life’s unpredictability and explained through three main concepts:

- *Middle consciousness* is an ability to create order. When you place situations that cannot be controlled into standby mode, they can be disconnected from your consciousness without being completely repressed.
- *Destiny readiness* is an evolved acceptance and adaptation to uncontrollable events. Instead of expecting life to be easy, safety is found in the manageability of each event.
- *Doing safety* means an active approach to life where people in communion with others create their own safety. It includes a flexible view on time.

In order to illustrate the move from problems to solutions, we have designed a model, inspired by Kaner (Kaner, 2014), illustrating the submissive patientification process and our alternative approach, which might empower and include patients as co-operative partners. The purpose of our study is thus to explore *if* and *how* Momentary contentment theory may provide solutions to issues experienced by cancer patients and their relatives in order to break the patientification process and move it towards co-creation of care.

## Materials and method

### Momentary contentment theory (a)

The data consists of interviews, conversations and notes from observations of everyday life in a remote village in northern Norway, from 2010 to 2014. The first author conducted a total of six unstructured and semi-structured focus groups and eight individual interviews that lasted between 2–6 hours each. In order to capture views of their everyday lives the informants were asked to freely talk about their experiences. The first author also gathered field notes from 15 conversations and 50 informal, semi-structured conversations. New decisions regarding data collection were made after each interview (Glaser, 1998). Notes from interviews and observations were written and theoretical memos were both written and drawn in different shapes and forms in the comparative process. These memos have been sorted, coded, categorized and compared to find relationships between categories and concepts using theoretical codes. After each interview or accrual observation the new material was coded, analysed and compared with previous results. Data was thus collected and analysed in stages until new data did not provide any new information, i.e., saturation was reached. At saturation the formulated theory was slightly modified in light of existing literature (Glaser, 1998). The analysis and methodology are further described elsewhere (Sandén, 2017; Sandén, Harrysson et al., 2015).

### Interviews with cancer patients and relatives (b, c)

The patient interview data is based on interviews with 36 participants affected by cancer, 19 patients (study b) and 17 relatives (study c) of cancer patients in southern Sweden. Ages were between 20 and 70 years, men and women. For ethical reasons we did not collect more personal data from the participants. All of the interviewed patients were considered cured or in disease remission. The represented cancer illnesses were acute myeloid leukaemia, head and neck cancer, oesophagus cancer, prostate cancer and bladder cancer. Among the relatives, cancers of pancreas, breast, bone, kidney, lung, CNS, lymphoma, myeloma and sarcoma were present. Dementia or major depression were exclusion criteria to participation. The interviews were unstructured and lasted 2–3 hours. In accordance with classic grounded theory (Glaser, 1998) no interviews were recorded, instead, detailed notes were taken during the interviews. The starting question was: “Please tell me about your lives”. At the end of some interviews we asked questions to confirm interpretations of previous analysis to avoid misunderstandings. Questions were similar to “What did you mean when you said … ?”

We started out with an analysis inspired by classic grounded theory resulting in two published studies (Sandén et al., 2017, 2019). No new fully integrated grounded theory was generated, but a main concern of navigating in a new and unknown life situation emerged regarding both patients and relatives. Different issues related to health emerged in the data (Table I) such as waiting, delegitimation (Ware, 1992), fear, hope, knowledge gaps, loneliness and health (Sandén, 2017).

**Table I.** Issues that emerged from the interviews with patients (b) and relatives (c)

	PATIENTS	RELATIVES
WAIT	Taught to passively hope for good results	Learning to hope for good results
DELEGITIMATION	Health care staff patronizing patients Patient’s body and life are fragmented	Hard to reach healthcare staff
FEAR	Death is always apparent	Fear of being left alone
HOPE	Learning a “hoping-for” state of mind	Hoping for good results
KNOWLEDGE GAPS	Difficulties to turn information into knowledge	Lack of information
LONELINESS	The disease creates feelings of loneliness	No one to talk to
HEALTH	A black-and-white view of being healthy or sick	Own health diminished behind the illness of the cancer sick

### Revisiting interviews with cancer patients and relatives through momentary contentment theory

We have previously (Sandén et al., 2017) shown a theoretical fit between the contexts of cancer patients and the subjective area from Momentary contentment theory. We therefore combined the two studies (study a and b) through a design-thinking approach, but from an inductive grounded theory base. For the present study, we have moved focus from an inductive grounded theory approach to a more deductive approach where the notes from interviews with the patients and relatives are interpreted through Momentary contentment theory.

We have also brought memos from our previous studies into the new analysis work, see example in Result section “Fear and ‘scanxiety’ (i.e., own health diminished behind the illness of the cancer sick): Waiting or preparing”. In our conceptualization of the patients’ stories, we searched for meanings of their whole life situations while moving between each need and concept, such as waiting, worries, relationships etc., and then we analysed the dialectical interaction between the concepts and health as a whole. An example: many patients and relatives expressed problems related to waiting. We did not see that issue in our Momentary contentment study (study a) data because the group studied solved the problem. Consequently, we reflected on how patients solved the issue of waiting and based on the reflections tried to illuminate different strategies and emergently fit these into the patient descriptions.

#### Multidisciplinary analysis

Our analysis is multidisciplinary and made possible through our different backgrounds. We represent medicine, social work and design engineering and have participated in the first analysis of the interviews (studies a and b) as well as in this re-analysis. First, second and third authors have participated in the emergence of Momentary contentment theory (study a). All interviews were conducted by the first author, with the last (fourth) author participating in two of the cancer patients’ interviews. The third author has been involved in the immediate analysis and conceptualization of the cancer patient interviews. By applying Momentary contentment theory to the concepts gathered from the interview narratives we introduce social medicine to a new health theory.

The regional ethics committee at Lund University approved the studies (Reg nr 2015:53 and 2016:219).

### Design thinking and the diamond of participatory decision-making

**Figure 1.**
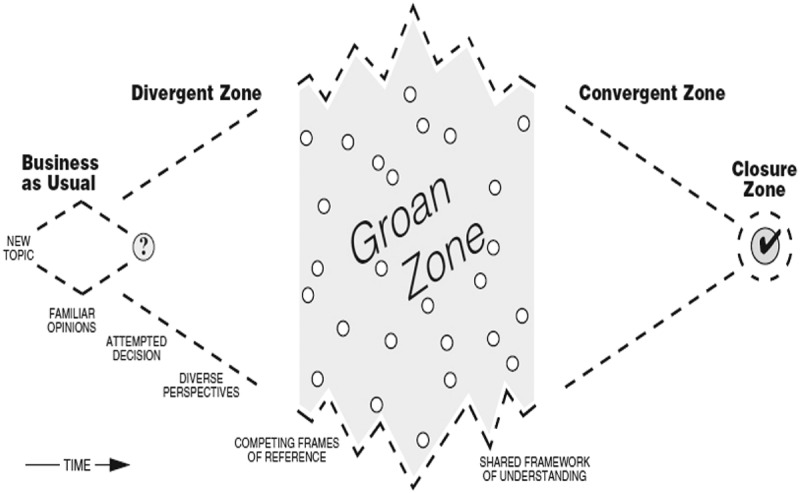
Diamond of participatory decision-making

The concept of shared decision-making has been proclaimed as a prime approach of making healthcare decisions since the early 1980s. However, its implementation is still a challenge due to organizational and cognitive gaps between service providers of healthcare and patients (Shay & Lafata, 2015; Weston, 2001). Bridging these gaps and including user experiences are major reasons for using design thinking for theoretical and practical guidance (Mintrom & Luetjens, 2016). Design thinking has evolved from creativity techniques and for the past 20 years has been popularized and used in various contexts to solve “wicked” problems by combining practical processes with cognitive and strategic dimensions of reasoning. A central theme in design thinking is to understand the user thoroughly and to use user experiences and interpretations in the creation of solutions. Correspondingly, when design thinking is applied to healthcare, analysis is initially based on the patient’s narratives for in-depth understanding of underlying patterns and needs. We use Kaner’s (Kaner, 2014) pedagogical model “Diamond of participatory decision-making” (figure 1) as a device to illustrate the change processes cancer-affected people may experience. The model shows the process of decision-making among different individuals and competences involving the different perspectives, frameworks and assumptions each part has in a group. In our context, this model is adjusted to include the different issues a cancer patient has to deal with.

In this original figure (Kaner, 2014) the model shows phases that groups go through when facilitating sustainable agreements.

In the divergent zone different perspectives become visible as a result of different expectations and assumptions, leading to competing frames of references when it comes to why, what, when and how healthcare is provided and consumed. The groan zone is per se a consequence of the diversity of perspectives, goals and knowledge that emerge in the interaction among people. Misunderstandings and miscommunications are seen as inevitable and normal in participatory decision-making (Shay & Lafata, 2015). It is through groan zones that different frames of reference meet in order to converge into a new shared frame of reference. The Diamond of participatory decision-making has been used before as an individual identity exploring tool by the first author in regard to brain rehabilitation (Sandén, 2019). Sevetson (Sevetson, 2005), refers to organizational changes while discussing a personal journey through the Diamond of participatory design, where there is personal pain in the groan zone.

## Results

### The patientification process

**Figure 2.**
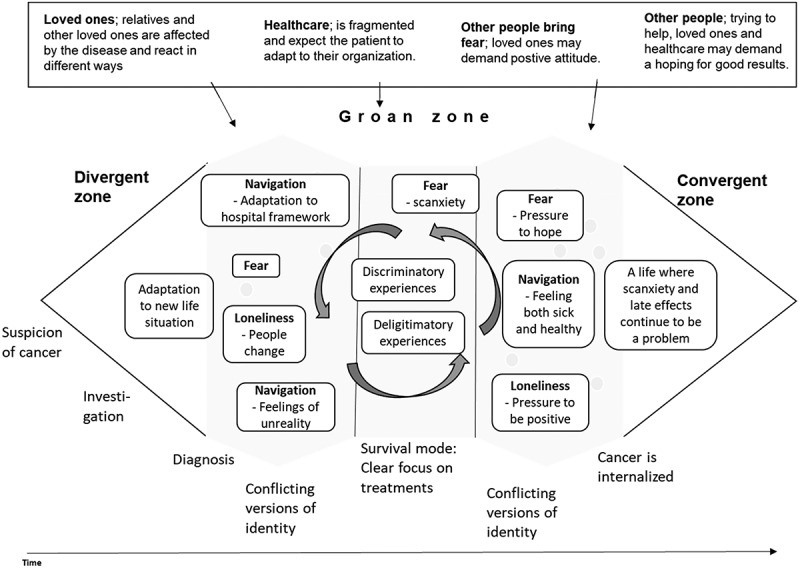
Patientification process

Our informants described cancer appearing in different steps, not as a straightforward process, but an iterative move towards a new life. Looking at the participatory decision-making model from a cancer patient identity perspective (figure 2), getting cancer is described by our informants as a divergent period where new perspectives are brought into their lives.

When a diagnosis is established, many patients describe confusion about whether or not they are ill; they describe it as unreal. They suddenly must involve healthcare in their everyday life and their social life changes. Studies involving 15 cancer survivors over 65 years of age show that disruptions to time and identity induce a biographical reconstruction (Hannum & Rubinstein, 2016). Other people change in the way they act towards the sick person and some relationships grow stronger; others disappear. The *groan zone* is described by our informants as three different subzones. In the first subzone the person tries to integrate the illness and its consequences into/with their identity, often expressed in relation to a shortened life span “*I have to live life, maybe all I have is this moment*”. Here several patients and relatives expressed being met with fragmentation into body parts: *“I became a stick figure”*. The first subzone of the groan zone is often described as a period of anxiety and disbelief.

Then, in the second subzone, when treatments start, patients are focused on surviving, and bodily reactions to treatments. Here the patients, but not the relatives, have intense contact with healthcare. The patient still must relate to their history, to the future and their relatives’ view of both what has happened, what is happening and what will happen. As one informant expressed it “*How can I understand a side effect before I have lived it?*” referring to both his bodily struggles and to difficulties getting others to understand his new situation.

In the third subzone the person tries to integrate the whole experience with life after cancer or with cancer. The *convergent zone* integrates all experiences and supposedly makes cancer an incorporated part of a patient’s identity; the process is seen in expressions such as “*I have started to think more about myself*” and “*I create my own space*”. However, our informants seemed to struggle with the concepts of health and illness. One focus group used more than one hour to discuss whether they were ill or healthy in times of remission. Other groups shared the difficulty of integrating both health and illness with their identity and many expressed difficulties having to choose between the two. This struggle is also apparent in patients’ expressions such as “*I am in a pretend-to-be-healthy mood*” or “*healthocondria*”. After treatments feelings swing between the unreal and a new life. The situation facilitates a complex incorporation in the identity process of the cancer patient. The data shows a patientification process where cancer patients go through different stages in an iterative process where they learn to be a submissive, patiently waiting patient. Other people are in control of their care and dependence, together with delegitimation experiences, pushes them towards a state of passivity. This is illustrated in figure 2, a design model based on the Diamond of participatory decision-making.

### Introducing momentary contentment to reduce patientification impact

**Figure 3.**
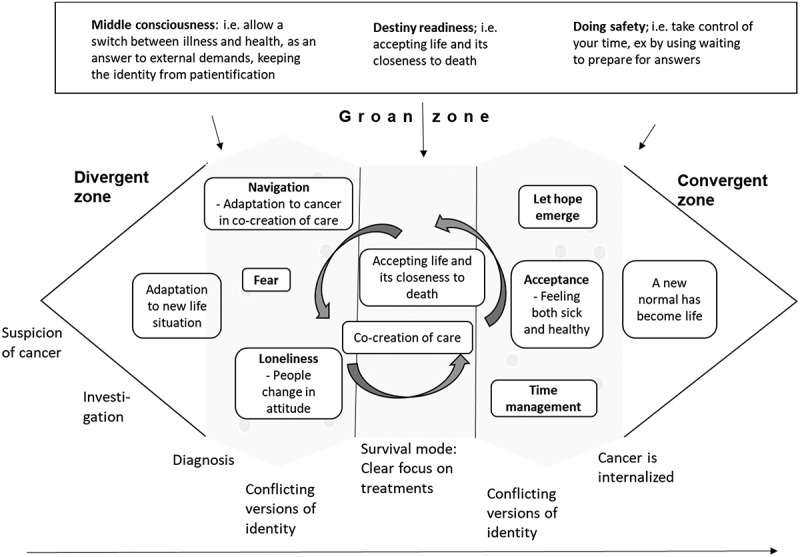
Momentary contentment process

Figure 3 illustrates how strategies illuminated by Momentary contentment theory supports staying in the moment with patients feeling fewer worries and increased hope.

Instead of patient passivity, we argue an alternative approach where danger is met with personal involvement, a clear continuum of care and activities that help one getting back to the moment. Using the main concepts of Momentary contentment theory, *Middle consciousness* forms intermissions in the moment. By placing thoughts and feelings like fear in a cluster of moments, or redirecting them towards black humour, a sense of safety can be established outside of that cluster of moments (Sandén, 2017). *Destiny readiness*, accepting life as hard, contributes to a feeling of a crisis as a normal situation that must be dealt with. Through helpfulness, collective safety structures and activity, a crisis is then managed. *Doing safety* shows the possibility to affect a personal situation of any kind. A study on community-based activity groups among the elderly shows that doing activities together with others enhances the positive effect of well-being as well as the motivation to keep going (Lindsay-Smith et al., 2019). Joint activities are partly a way to help each other, are partly therapy, but are also the means to have fun. The joint activities give a sense of control to do something as it cognitively creates clusters of moments, controllable episodes within an activity. With Momentary contentment theory a deeper understanding of the interview data could be gained, as shown in Table II.

**Table II.** Combining concepts from patient narratives and Momentary contentment theory

	What people said during interviews	Middle consciousness	Destiny readiness	Doing safety
Wait	Takes energy Worrisome Hard to focus on anything else Living in the future	A mindset where waiting is put in standby mode	Knowing that “shit” does happen sometimes	Preparation Doing things as distraction
Delegiti-mation	Body was fragmented, as well as body and soul were separated.	A mindset where health professionals are allowed to be Godlike “he/she will save me” and also humans as everybody else	Preparing for “shit” happens also when meeting healthcare professionals	Health professionals must learn to meet people as human beings with body, mind and soul
Fear	Stands in the way of health. Comes and goes with scanxiety	Allow hope to grow while you are afraid	Every time you become aware of having survived, hope grows	Meet fear with activity. Preparation and distraction
Hope	Important Must be logical	Hope is a state of mind	The knowledge of “shit happens” moves hope from a future good result to a calmness that you can handle anything	Doing things which help keeping mind and thoughts in the moment
Knowledge	Hard to go from informed to knowledgeable	Knowing you can handle anything creates hope	Experience-based	Learning
Loneliness	The disease creates loneliness. It is great to meet other cancer patients	Meeting others in the same situation allows life to be as it is, and no words are needed. You can stay in the middle consciousness without denying reality	Meeting others who know things can happen makes it less lonely. Reality is allowed	Reaching out to other people
Health	A black and white pendulum between feeling ill or feeling healthy	Allowing for the self to be both ill and healthy at the same time	Learning about the disease and accepting it as a part of the body	Doing new things, exploring life

### Fear and “scanxiety”: waiting or preparing

#### The distortion of time through waiting

Risks of a shortened future create emphasis on the current moment; “*I have been given a chance to rethink what is really important to me*”. However, our data also shows how patients frequently put their lives on hold while waiting for answers. Time, inflicted as waiting, may disempower patients, “ … especially to be kept waiting an unusually long time is to be the subject of an assertion that one’s own time (and therefore, one’s social worth) is less valuable than the time and worth of the one who imposes the wait” as Schwartz (Schwartz, 1974) (p, p. 856) states. This negative situation may be balanced through medical consultation. An informant expressed this in different words: *“I had a great physician; he was calm and seemed to have all the time in the world. We talked about other things also and he told funny stories”*. The opposite was also expressed, where both healthcare staff and relatives frequently tried to reduce the distress with expressions like “don’t worry” and “let’s hope for the best”. This may lead to two problems:

- Delegitimation. In our interviews patients talked about hypochondria when scared, thus making an adequate feeling pathological. Such expressions also cause feelings of demands being made; *“I will kick the next person who asks me to be positive”.*

- Passivity. Just as the population studied in the Momentary contentment theory study tie down their outdoor furniture before an expected storm, cancer patients can create safety by preparing for different results. Telling patients not to worry encourages them to passively wait.

Further, these expressions tend to focus on the future, when scan results will be available, away from the moment, where a patient can actually *do* something about their situation. Life becomes a “negative journey” accompanied by “scanxiety”, which starts a while before the scan, and persists until scan results arrive. Our informants describe how they do not know what will happen after they receive the scan results. With proper preparations they may be able to relax more and regain some control. *Waiting for imaging result. Timeline of “Scanxiety”, distress reported by patients scheduled for diagnostic imaging to assess disease status. The condition, “scanxiety”, is linked to decreased quality of life (*Bauml et al., 2016; Portman, 2018).

Our informants often chose to divide life into “being ill” while waiting, and “being healthy”, as in survival. They showed a linear perception of time during waiting (Figure 3) and described a “hoping-for” state of mind, where hope was placed in the results. Living with hope, on the other hand, allows you to cope: “*While waiting for an X-ray result, I’m afraid, otherwise I don’t think that much about it*”.

### Hope, time management and knowledge

#### The distortion of hope

For cancer patients, hope is closely connected to waiting. Being hopeful relates to feelings about what is in the present and hoping for something is related to change and the future (Benzein, 1999; Benzein et al., 2001). There is thus a difference between living with hope and hoping for something. Living with hope means an acceptance of life and a belief that you can handle future challenges.

**Figure 4.**
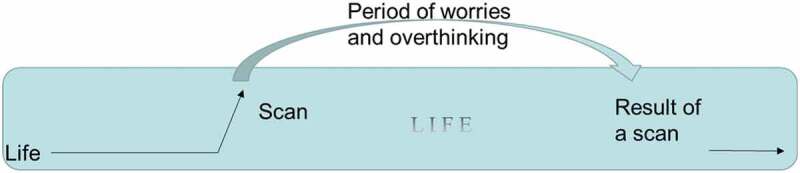
Illustration of “Scanxiety”

With threat of a shortened life, healthcare professionals as well as relatives often try to enhance hope, which can lead to increased suffering (Törnqvist & Nielsen, 2011). The need to hope for a good result pressures people to cognitively move from the moment to an uncertain future. It also promotes a passive stance towards the results and the upcoming types of care taking activities. Waiting and “hoping for” are connected in their denial of the momentary reality. “Let’s hope for the best” risks reducing a patient’s hope by precluding activity as well as moving focus towards a worrisome future. See figure 4.

In order to allow for an incorporation of offered hope, not making it feel forced, the hope aimed for must match the knowledge a patient has. As one informant said: *“I just got angry when they obviously lied to me since I had read the [bad] statistics”*.

### Loneliness and companionship

In this article we show how patients are fostered and even forced into a patient identity (Figure 2). Gunnarson (Gunnarson, 2016) discusses how becoming a patient involves a transformation process from being a subject, to one’s body becoming an object. One informant said: “*I walk into the hospital as a human being but walk out as a jaw*”.

There are many studies on the relationship between physicians and patients. A study on identity construction of medical students shows how students grew to connect physicians as allies and patients as counterparts; some saw patients as adversaries (Warmington & McColl, 2017). This was also detected in our interviews from a patient perspective, where one patient expressed the feeling when being treated in a condescending way: “*you and I do not belong to the same kind of people*”. Another patient expressed feeling offended when talking about radiation side effects and the physician answered “*no, you don’t experience side effects, they don’t come until next week*”.

Patients are told not to worry, when they in fact are dealing with a possibly deadly disease.

The cancer patients we interviewed expressed a need to be taken seriously when asking healthcare for help. If that did not happen, patients risked falling into self-doubt. Such self-doubt was hidden in different ways in the interview material. One was in the difference between patients and relatives, where both groups had criticism and examples of where they had not been heard:

*“I wanted to be seen, not just the tumour”.*

*“No one took my symptoms seriously”.*

However, as soon as criticism was put on healthcare staff the patient informants continued adding something positive to counteract a negative critique or shared experience:

*“but I shouldn’t complain, they saved my life”.*

Whereas the relatives seemed genuinely disappointed:

*“It’s a lonely struggle to deal with the healthcare system”.*

The self-doubt was also seen in how several patients described having had symptoms for years, suspecting cancer, but, not only, accepting a physician’s word about the symptoms not being dangerous, but also making a hypochondriac comment about themselves, sometimes in combination with trying to avoid the risk of getting labelled as such. Similarly, Nordenfelt (Nordenfelt, 2005) discusses the importance of patient communication with their medical staff and argues that lack thereof may create feelings of insult and humiliation in a chronically ill person. This was also true for the relatives in our interviews. Several of them expressed how they thought they became mentally ill due to anxiety in the stressful situations where they had nowhere to turn. The stress of seeing a relative become more and more ill also created a horror-like situation where they wanted to help but did not know how. The frustration made many relatives hope “for it all to end”, a hope that risked leading to guilt since the person suffering from cancer might not survive. All these mixed feelings together with the uncertain future affected a relative’s identity and many informants expressed relief when meeting people in the same situation: *“I realized that I am not alone, that I am not crazy, that one may actually feel like I do”*. The situation was often unbearable for relatives and involved a high level of frustration. This was something the person suffering from cancer needed to relate to. As a result, both parties were at risk of assuming feelings of guilt and shame. This may explain some of the loneliness patients and relatives experience when a family member is ill, and why meeting others in shared situations provides important benefits. Several studies show that both cancer survivors and their relatives suffer from stress and depressive symptoms (Han, 2017; Lin et al., 2018; Osowiecka et al., 2019; Sandén, 2017; Sjövall, 2011). Important to note, however, is how patient informants described feelings of safety when they had been able to keep the same healthcare staff over time.

## Discussion

In this article we revisit earlier used data from interviews with cancer patients and relatives where, in a deductively inspired analysis, we add Momentary contentment theory. In this re-analysis of qualitative interviews, we have found that a patientification process where patients are taught to be submissive may be reduced by a Momentary contentment approach. This includes a shift from patientification to co-creation of care. Both cancer patients and relatives to cancer patients expressed themselves in relation to each other in interviews, and both groups also emphasized the importance of their relationships, or lack thereof, with healthcare staff. Heaven et al. showed how trial identity shapes participants’ understandings regarding treatment decisions and all other aspects of the trial (Heaven et al., 2006). People learn about expected values and practices, including how trial group members are expected to treat one another (Lave & Wenger, 1991). Even though identity is an individual marker it is thus formed and reinforced in relationships. The informants lift themselves in relation to the health professional team: *“no one wants to be labelled a difficult patient”.*

### Patientification process

It is within relationships that patientification is strengthened and weakened. If we look at cancer patients as belonging to a cultural group within healthcare, their view of themselves will be affected when they are objectified as patients. Prejudice and discrimination are complex social phenomena negotiated through an intricate interactional web that involves initiation from the dominant group and definition and reaction from the subordinate one (Evergeti, 2011). Parsons (Parsons, 1951) discusses illness as more than a condition, as a social role, the sick person role, where the person is deprived of a reasonable claim of full legitimacy. In a patient role they are obliged to accept help from those who are specially qualified to treat illness, mostly physicians. It seems that our embodied experiences change when we become ill (Gunnarson, 2016). The fragmentation was evident in our interviews and one patient described how she lost her sense of being a human: *“I became a stick figure”*. This resembles Agledahl’s (Agledahl et al., 2011) study where physicians, often without realizing, ignore existential questions. Ware discusses delegitimation and mentions two types: one where people minimize the experience of illness with words like “*we are all tired*”, and another where physicians define the experienced illness as existing mostly in the patient’s mind, i.e., a psychosomatic illness (Ware, 1992). Both types mean a questioning of a person’s own experiences, however, according to Ware, the second one is more damaging to the patient since it includes a new illness, a psychological one, which contains a great deal of stigma. The expressions “*let’s hope for the best*” and “*don’t worry*” both risks contributing to delegitimation. In France, the concept of “Patientilisation” has been used to explain a patiently waiting patient (Petter-Zaugg, 2013). This correlates to the concept of clientification in social work, which includes a categorization process where a problem gets defined within the organizational frame, often in a landscape of fragmentation, specialization and individualization (Gümüscü et al., 2015). Translated into healthcare we can say, in a similar fashion, that there is a patientification process in progress.

### Regaining control of your temporal space

According to Gadamer, a main task for healthcare is, in addition to restoring the sick person, and in connection with recovery, to reproduce unity with self (Gadamer, 1996). New living conditions require new social constructs, and to regain everyday life you must accept and adapt to new living conditions. Previous research on cancer patients illustrates their difficulties in balancing their new abilities where fatigue and other late effects have become a part of life, with both internal and external demands (Berger et al., 2015; Duijts et al., 2017; Van Maarschalkerweerd et al., 2019). Working full-time becomes difficult. A study of breast cancer survivors in the Netherlands shows changes in employment status 5–10 years after diagnosis (Van Maarschalkerweerd et al., 2019). According to Momentary contentment theory the view on time management and life priorities needs to be adapted to current situation (Sandén, Thulesius et al., 2015). The anthropologist Alfred Gells describes how several human periodization’s have their origin in different natural phenomena that are not socially determined, such as the year and day, which unlike the socially constructed week and hour, are based on the Earth’s relation to the sun (Heidegren, 2014). Likewise, we can choose to allow disease to create new accruals, which will be more adapted to real life. A natural, flexible and compliant view of time would both accept feelings of fatigue due to treatments and of joy in times of health.

By relating to the present moment as a subjective experience, not following clock time, it differs from situation to situation and between people. Life and its requirements can be adapted to a new life situation, which includes illness. Instead of the paralysing wait, patients may participate in preparations for different possible outcomes (figure 4). In Momentary contentment every moment has its own context and as such creates a possibility to influence the context in which the next moment will take place. Activity may bring a patient from a “hoping-for” state of mind to a present “living with hope”. In the process patients become more involved in their care, which in several studies has shown to be beneficial (Alden, 2014; deBronkart, 2011; Kane et al., 2014; McDonald et al., 2013; Schmidt et al., 2015).

**Figure 5.**
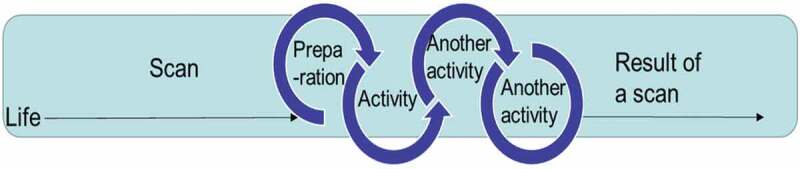
Overcoming Scanxiety

Figure 5, Overcoming “Scanxiety”. By creating a cyclical perception of waiting through preparing activities, anxiety-provoking anticipation may be reduced.

Memo on overcoming scanxiety through Momentary contentment theory:

*Through preparation you create a feeling “I have done everything I can do”. To be able to do so you need knowledge. In preparation hope emerges, “I*do*handle this”. Every time you handle the situation you become more assertive that you can handle whatever comes in your way. Different outcomes during waiting become apparent when you are preparing and when they are all prepared for it is hard to dwell on them. When everything possible is done there is not much to do but to focus on something else; like having fun. That can be hard due to the loneliness, the lack of understanding. Everyone understands that you may be afraid, but it may be harder to understand your calmness. Here it helps to find others in similar situations and perhaps with similar personalities. Meeting others in the same situation allows life to be as it is and no words are needed. You can stay in middle consciousness without denying life as is.*

### From patientification to participation and co-creation of care

Gadamer (Gadamer, 1996) sees health as personal, an independent non-measurable balance. Antonovsky stresses the balance between generalized resource deficits and resources to determine whether something will be harmful or not (Antonovsky, 1987). This resembles Momentary contentment theory (Sandén, Harrysson et al., 2015) where health is found in the balance between the dangers in an arctic climate and the villagers’ ability to handle them. Momentary contentment theory adds activity, an acceptance of different outcomes in life and a stand-by mentality (figure 5), which makes the theory usable as an antagonist to the passivity surrounding the patientification process.

It seems as patients find a way to live with hope even when the situation looks bad from the side-line. Momentary contentment theory (Sandén, 2014) illustrates an incorporation of different aspects of life as normal, including accidents and illnesses, which facilitates an internal readiness. That readiness creates a hope with no destination or change, a hope that lives within. Contrary to “hoping for a good result” this intrinsic hope moves people from anxious thoughts about what may happen in the future to a momentary acceptance of life as unpredictable and hard to control. Safety is instead reached through activity, cognitive awareness and collective helpfulness. One such activity is predictability. Together with relevant knowledge of different processes you can prepare for different results. One man stated how he managed to cope by focusing on getting well due to the information he was given about the importance of the first month “*I thought I will give everything these 30 days*”. Our informants expressed the importance of not thinking of the disease when feeling healthy, stated in “*when waiting I am sick, otherwise I don’t think about the disease*”.

When you have cancer, it is hard to feel in control. Yet through preparations, a form of activity, a sense of control can be achieved. Activities can further be used in companionship with others to bring someone back from a negative moment, as shown in breast cancer dragon boat participants (McDonough et al., 2019). An Australian study shows how physical exercise improved both somatic and mental health among cancer survivors (Frensham et al., 2018) and a case study with exercise rehabilitation in a glioblastoma patient shows quality of life improvements (Hansen et al., 2019). To many people cancer becomes a life-long experience. Through an acceptance that different outcomes are possible one may start to prepare. In the preparation lies a subjective sense of control. Through the combination of activity and a feeling that everything that *can* be done *has* been done, patients may feel healthy in the moment, without denying the disease.

### Implications for future studies replacing the patientification process with co-creation of care

In this section we have theorized our results and the implications are to be seen as suggestions that have not yet been empirically studied. We suggest these to be implemented during a clinical study. Principles for breaking the patientification process for staff in personalized cancer care are:

* Focus on each person as an individual with both unique and common needs. This supports the patient’s feeling of being included in their own care.

* Support knowledge development. It strengthens the patient.

* See to that the person is included into shared decision-making. To do so the principles above must be regarded. Together they obstruct the patientification process.

* Help patients and relatives to distinguish between hoping for something and living with hope.

* Activity may be used both to prepare for different outcomes as well as to distract from passive and anxious waiting.

#### Strengths and limitations

Our secondary analysis of revisiting data initially collected for another analytical purpose asks for some caution. Thus, we have been observant about data that may not entirely fit the theory of Momentary contentment. We have both gone back to the raw data and to our earlier interpretations to check the data integrity against the eventuality that our new analysis may have changed the meaning of what informants shared with us. The analysis presented in this article does not contain any skewed data to create perfect fits although a grounded theory should be modifiable when new data are analysed. Hence, it may be possible that “no-fits” were left out.

The data itself also holds some limitations. Our interviews were conducted with patients and relatives after cancer survival or death, so that the narratives are constructions of memories. The interviews were conducted by one author. They were not recorded, instead, detailed notes were taken in accordance with classic grounded theory. To overcome possible bias, we have had one other author participate in two interview sessions to see how notes may differ. The differences were minor, and we regard them as having no impact on our analysis. A second way to credibility-test the data was by sending the analytical work to the participants for comments. The feedback we received confirms our data interpretations. The interview data has also been discussed among the authors, all having experience in working or living with cancer patients.

The Momentary contentment theory has not been built around, or tested empirically on, cancer patients. However, what we have done is to present a conceptual design through theorizing needs and solutions based on conceptualized cancer interview data. There is a multidisciplinary approach in our analysis, based on the research group’s various disciplines where we mix engineering, medicine and social sciences as well as clinical and patient experiences.

## Conclusion

Through Momentary contentment theory we introduce participation and acceptance as means to learn to adapt to new living conditions for patients with cancer. We suggest a clinical study where patients are guided into a proactive approach to concepts such as living with hope, activity, preparation and acquiring knowledge. Moreover, healthcare is assumed to be a co-creation process, including the patient based on their needs.
